# Inorganic Materials and Metal–Organic Frameworks: Editorial Announcement

**DOI:** 10.3390/nano11123279

**Published:** 2021-12-03

**Authors:** Félix Zamora

**Affiliations:** Departamento de Química Inorgánica, Universidad Autónoma de Madrid, C/ Tomás y Valiente no. 7, 28049 Madrid, Spain; felix.zamora@uam.es

Dear Readers,

The great science from our authors and the high support our readers have positioned ”Nanomaterials” as a top scientific journal with a continuous impact factor increase. We also have an excellent group of reviewers supporting the journal that actively enhances the journal’s quality—the increasing trajectory of the journal results in the current impressive impact factor of 5.076. I must congratulate our Editor-in-Chief Prof. Dr. Shirley Chiang, for these outstanding results.

From mid-2019, we started a new section in Nanomaterials focused on “Inorganic Materials and Metal–Organic Frameworks”. This section covers a multidisciplinary science at the frontier between nanomaterials and inorganic chemistry. It focuses on different types of materials, including monoelemental and multicomponent nanoparticles with different shapes and sizes and incredible physico-chemical properties, enhancing applications to atomically designed particles. We also include a particular sub-section on metal-organic frameworks because processability at the nanoscale of these porous and crystalline materials has recently become a topic of increasing scientific attention due to their potential implication in many technological fields of application. The section has been very well received, publishing a total of 38 works of excellent quality in 2020 by scientific groups worldwide. Just to mention some examples of the most relevant contributions selected based on the number of citations, I would like to highlight a research article from Prof. Clement et al. (Germany) focused on Biocompatible Magnetic Fluids [1]; a contribution from Prof. Caballero (Spain) et al. centered on a Metal–Organic Framework valuable as cathode for long-cycle Li-S batteries [2]; and a review article from Prof. Djordjevic et al. (scientists from Czech Republic and Serbia) analyzing properties, application, and toxicity of Carbon Nanomaterials [3]. We do not doubt that this excellent start is just a preview of what is to come.

It is a great honor for me to follow at the position of Inorganic Materials and Metal–Organic Frameworks Section Editor-in-Chief of Nanomaterials.

We are excited about the future. We want to support the publication of the scientific works in fundamental and applied science, dealing with preparation, characterization, properties, device fabrication, and applications to theoretical studies to enhance materials design and properties.

The Inorganic Materials and Metal–Organic Frameworks section is currently organizing 22 Special Issues covering the most fundamental aspects of the research in inorganic materials at the nanoscale. Some of these issues are led by world-leading scientists or rising stars. For example, Prof. Wang of The Chinese Academy of Sciences is leading a special Issue on Advanced Metal–Organic Frameworks. At the same time, the young scientist Dr. Marius Dobromir acts as guest editor of the Special Issue “Growth, Characterization and Applications of Nanotubes”.

We look forward to working with the researchers, reviewers, and the brilliant editorial staff of *Nanomaterials* to continue to keep on the journal’s quality. Our goal is to publish a variety of high-quality, high-impact research on inorganic nanomaterials.

With best regards,

Félix Zamora
